# Supplementary material for: Empirical methods for controlling false positives and estimating confidence in ChIP-Seq peaks
Source: BMC Bioinformatics. 2008 Dec 5;9:523. doi: 10.1186/1471-2105-9-523 (PMC2628906; doi:10.1186/1471-2105-9-523)
Supplement: Additional File 1 — A variety of html documents from the USeq web site detailing the available applications, their best usage, output file type descriptions, command line menus, etc. [file 1471-2105-9-523-S1.zip › USeqUserGuides/JavaDoc.zip!/JavaDoc/trans/main/WindowBlockMakerTwoColor.html]

WindowBlockMakerTwoColor


|  |  |  |
| --- | --- | --- |
| **Overview** \| **Package** \| **Class** \| **Use** \| **Tree** \| **Deprecated** \| **Index** \| **Help** | |  |
| **PREV CLASS**   **NEXT CLASS** | **FRAMES**    **NO FRAMES**     **All Classes** |  |
| SUMMARY: NESTED \| FIELD \| CONSTR \| METHOD | DETAIL: FIELD \| CONSTR \| METHOD |  |


---


## trans.main Class WindowBlockMakerTwoColor

```
java.lang.Object
  trans.main.WindowBlockMakerTwoColor
```

---

``` public class WindowBlockMakerTwoColor extends java.lang.Object ```

Converts a Window[] into a heat map specific bar file for import into IGB.
Assumes windows are sorted by chromosome and start position
Score index is the index to use when fetching a score for a window from it's double[] of scores
see ScanChip or ScanChromosomesCNV for current score indexes.
Note, this will place at the 0 and 1 base two values to maintain symetry in the values.

---

**Constructor Summary**

`WindowBlockMakerTwoColor(int sizeOfOligo,
java.lang.String genomeVersion,
java.lang.String strand,
double minWindowFilter,
double maxWindowFilter)`

`WindowBlockMakerTwoColor(java.lang.String[] args)`


| **Method Summary** | |
| --- | --- |
| `void` | `add(int base, float value)`             Adds a gr line to the global ArrayLists for later burning to bar. |
| `void` | `addHeatMapBlocks(int startIndex, int stopIndex)`             Make a heatmap blocks from given window indexes, stop not included. |
| `void` | `assembleHeatMapBlocks()` |
| `void` | `balanceValues()` |
| `Window[]` | `filterWindows(Window[] windows)`             Tosses any windows within the min max filter range. |
| `double` | `getMaxWindowFilter()` |
| `double` | `getMinWindowFilter()` |
| `static void` | `main(java.lang.String[] args)` |
| `void` | `makeHeatMapBarFiles(Window[] windows)`             Makes a stair step heat map from an array of windows in bar format. |
| `void` | `makeHeatMapBarFiles(Window[] windows, java.io.File barDirectory, int scoreIndex)` |
| `boolean` | `overlapOrAbut(Window left, Window right)`             Assumes right window is to right of left or abuts or doesn't overlap. |
| `static void` | `printDocs()` |
| `void` | `printSgrLines()` |
| `void` | `processArgs(java.lang.String[] args)`             This method will process each argument and assign new varibles |
| `void` | `setBarDirectory(java.io.File barDirectory)` |
| `void` | `setMaxWindowFilter(double maxWindowFilter)` |
| `void` | `setMinWindowFilter(double minWindowFilter)` |

**Methods inherited from class java.lang.Object**

`equals, getClass, hashCode, notify, notifyAll, toString, wait, wait, wait`

**Constructor Detail**

### WindowBlockMakerTwoColor

```
public WindowBlockMakerTwoColor(int sizeOfOligo,
                                java.lang.String genomeVersion,
                                java.lang.String strand,
                                double minWindowFilter,
                                double maxWindowFilter)
```

---


### WindowBlockMakerTwoColor

```
public WindowBlockMakerTwoColor(java.lang.String[] args)
```


**Method Detail**

### makeHeatMapBarFiles

```
public void makeHeatMapBarFiles(Window[] windows,
                                java.io.File barDirectory,
                                int scoreIndex)
```

---


### filterWindows

```
public Window[] filterWindows(Window[] windows)
```

:   Tosses any windows within the min max filter range.

---


### makeHeatMapBarFiles

```
public void makeHeatMapBarFiles(Window[] windows)
```

:   Makes a stair step heat map from an array of windows in bar format. One per chromosome.
    Don't forget to set the barDirectory and score Index!!!!!!!

---


### balanceValues

```
public void balanceValues()
```

---


### printSgrLines

```
public void printSgrLines()
```

---


### assembleHeatMapBlocks

```
public void assembleHeatMapBlocks()
```

---


### addHeatMapBlocks

```
public void addHeatMapBlocks(int startIndex,
                             int stopIndex)
```

:   Make a heatmap blocks from given window indexes, stop not included.

---


### add

```
public void add(int base,
                float value)
```

:   Adds a gr line to the global ArrayLists for later burning to bar.

---


### main

```
public static void main(java.lang.String[] args)
```

---


### overlapOrAbut

```
public boolean overlapOrAbut(Window left,
                             Window right)
```

:   Assumes right window is to right of left or abuts or doesn't overlap.

---


### processArgs

```
public void processArgs(java.lang.String[] args)
```

:   This method will process each argument and assign new varibles

---


### printDocs

```
public static void printDocs()
```

---


### setBarDirectory

```
public void setBarDirectory(java.io.File barDirectory)
```

---


### getMaxWindowFilter

```
public double getMaxWindowFilter()
```

---


### setMaxWindowFilter

```
public void setMaxWindowFilter(double maxWindowFilter)
```

---


### getMinWindowFilter

```
public double getMinWindowFilter()
```

---


### setMinWindowFilter

```
public void setMinWindowFilter(double minWindowFilter)
```


---


|  |  |  |
| --- | --- | --- |
| **Overview** \| **Package** \| **Class** \| **Use** \| **Tree** \| **Deprecated** \| **Index** \| **Help** | |  |
| **PREV CLASS**   **NEXT CLASS** | **FRAMES**    **NO FRAMES**     **All Classes** |  |
| SUMMARY: NESTED \| FIELD \| CONSTR \| METHOD | DETAIL: FIELD \| CONSTR \| METHOD |  |


---
